# Perceval Induces Thrombocytopenia: Yes, of Course, but can we Change the Paradigm?

**DOI:** 10.21470/1678-9741-2020-0420

**Published:** 2021

**Authors:** Ignazio Condello, Giuseppe Santarpino

**Affiliations:** 1 Department of Cardiac Surgery, Anthea Hospital, GVM Care & Research, Bari, Italy.; 2 Paracelsus Medical University, Nuremberg, Germany.; 3 Department of Experimental and Clinical Medicine, Cardiac Surgery Unit, “Magna Graecia” University of Catanzaro, Italy.

Since the first studies published in 2011, the Perceval prosthesis has been associated with postoperative thrombocytopenia^[1]^. These studies originated from the association between this prosthesis and the previous model, Freedom-Solo, because both are constructed with the same biological material and because this previous model has "known" thrombocytopenic potential. Although studies in the literature have simultaneously shown that this thrombocytopenia has a transitory character and does not have a true clinical relevance, the "media" impact of this phenomenon poses a continuous debate on the origin of this event. Within this debate, we read the article by Mujtaba SS et al.^[2]^ who compared isolated aortic valve replacement (AVR) patients with sutureless Perceval S valve (Group A: 72 patients) and patients who underwent isolated sutured AVR with Perimount Magna Ease Bioprosthesis (Group B: 101 patients) between June 2014 and January 2017.

**Fig. 1:**
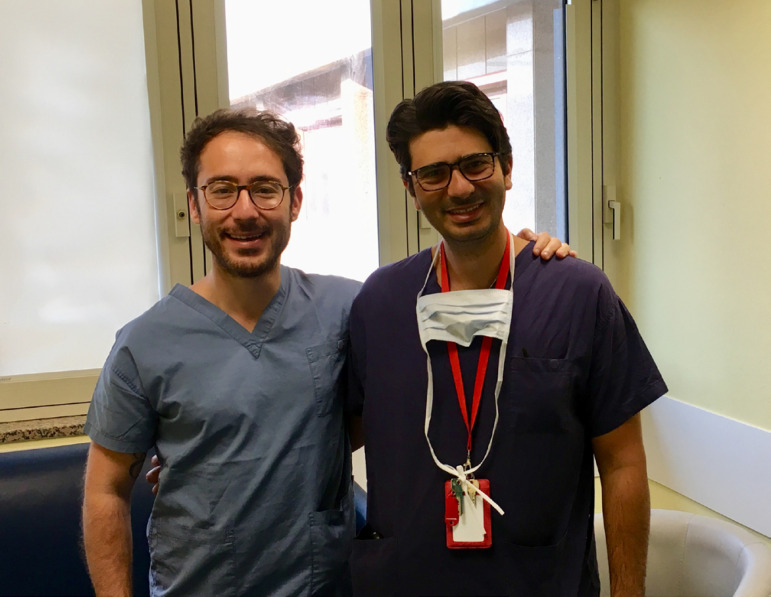
Ignazio Condello (on the left) and Giuseppe Santarpino (on the right).

These authors warn against the use of Perceval prosthesis because they concluded that with this model the risk of thrombocytopenia is significantly higher. In our opinion (Figure 1), this article needs some considerations for an "overall and integrated" choice of the prosthetic model to be used. The first consideration is that the recorded transient thrombocytopenia has no clinical impact, according to their own conclusions. Conversely, the use of Perceval prosthesis allows the implantation of a significantly larger prosthesis - therefore, providing a better hemodynamic duration over time - than other prosthetic models used. Therefore, there is a transitory and clinically irrelevant "disadvantage" *vs*. a significant long-term "advantage" in hemodynamics (= patient's quality of life). In addition, these authors concluded that the implantation of sutureless Perceval aortic valves was associated with a significant drop in platelet count postoperatively, with slow recovery and higher platelets and packed red blood cells transfusion requirements. Although this phenomenon, regardless of the theory that explains its origin, do exists, we believe that we could reason on how to associate the advantages of this prosthesis (*e.g*., stentless profile, ease to implant) with protective techniques on the platelets that allow "savings", by making the Perceval implant procedure "minimally invasive" and not only due to a “small surgical incision” (*e.g*., association with minimally invasive extracorporeal circulation).
